# Carbon Nanotubes: An Emerging Drug Carrier for Targeting Cancer Cells

**DOI:** 10.1155/2014/670815

**Published:** 2014-04-24

**Authors:** Vaibhav Rastogi, Pragya Yadav, Shiv Sankar Bhattacharya, Arun Kumar Mishra, Navneet Verma, Anurag Verma, Jayanta Kumar Pandit

**Affiliations:** ^1^School of Pharmaceutical Sciences, IFTM University, Moradabad, Uttar Pradesh 244001, India; ^2^Department of Pharmaceutics, Institute of Technology, Banaras Hindu University, Varanasi, Uttar Pradesh 221005, India

## Abstract

During recent years carbon nanotubes (CNTs) have been attracted by many researchers as a drug delivery carrier. CNTs are the third allotropic form of carbon-fullerenes which were rolled into cylindrical tubes. To be integrated into the biological systems, CNTs can be chemically modified or functionalised with therapeutically active molecules by forming stable covalent bonds or supramolecular assemblies based on noncovalent interactions. Owing to their high carrying capacity, biocompatibility, and specificity to cells, various cancer cells have been explored with CNTs for evaluation of pharmacokinetic parameters, cell viability, cytotoxicty, and drug delivery in tumor cells. This review attempts to highlight all aspects of CNTs which render them as an effective anticancer drug carrier and imaging agent. Also the potential application of CNT in targeting metastatic cancer cells by entrapping biomolecules and anticancer drugs has been covered in this review.

## 1. Introduction

**Figure 1:**
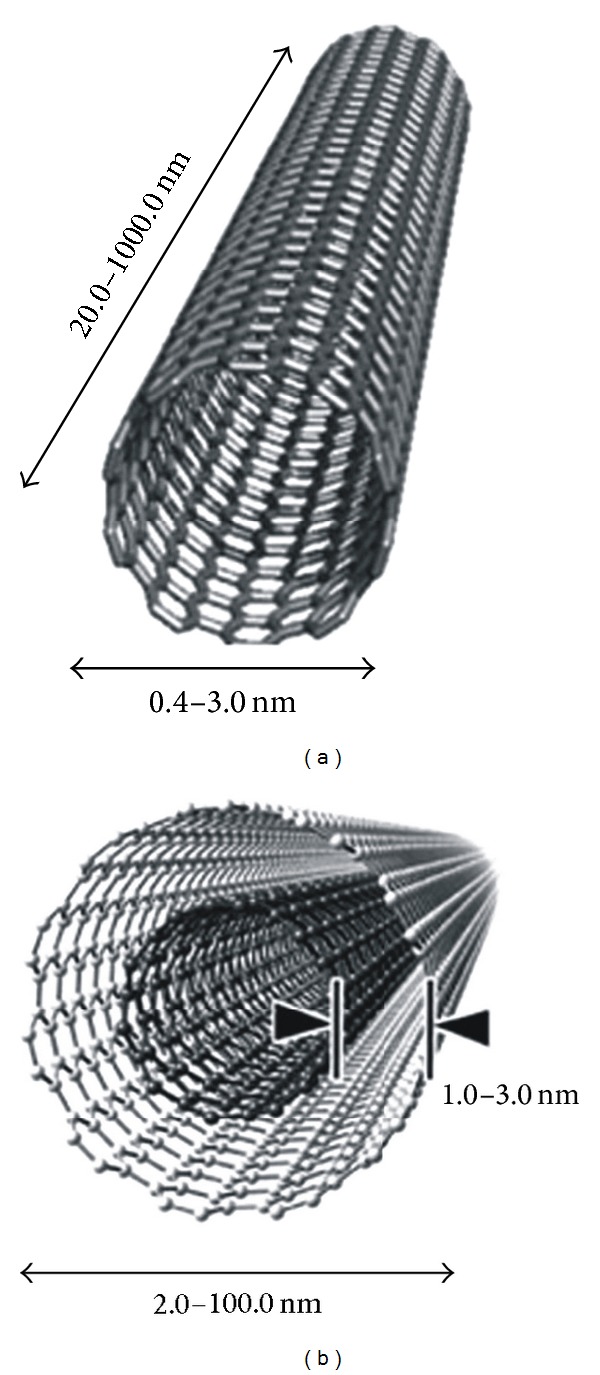
Carbon nanotube: (a) single walled carbon nanotube and (b) multiwalled carbon nanotube.

After the discovery of the third allotropic form of carbon fullerene in 1991, Sumio Iijima identified a new structural form of this allotrope, the cylindrical fullerene and named them as carbon nanotubes (CNTs) [1]. CNTs are graphene sheets rolled into a seamless cylinder that can be open ended or capped, having a high aspect ratio with diameters as small as 1nm and a length of several micrometers [2]. Depending on the number of sheets rolled into concentric cylinders, there are two broad categories of CNTs, namely, single-walled carbon nanotubes (SWCNTs) and multiwalled carbon nanotubes (MWCNTs) (Figure 1). SWCNTs are made up of single graphene layer wrapped into a hexagonal close-packed cylindrical structure whose diameter varies from 0.4 to 3.0 nm and length ranges from 20 to 1000 nm and are held together by Vander Waals forces, which makes them easily twistable and more pliable [3]. SWCNTs are produced by the electric arc [4], laser ablation [5], chemical vapor deposition (CVD) [6], and gas-phase catalytic processes (HiPco or high-pressure CO conversion) [7].

MWCNTs consist of several coaxial cylinders, each made of a single graphene sheet surrounding a hollow core. The outer diameter of MWCNTs ranges from 2 to 100 nm, while the inner diameter is in the range of 1–3 nm, and their length is 1 to several *μ*m [8]. Electric arc [9] and chemical vapor deposition (CVD) [10, 11] are the main techniques for their production. Owing to the sp^2^ hybridization in MWCNTs, a delocalized electron cloud along the wall is generated which is responsible for the *π*-*π* interactions between adjacent cylindrical layers in MWCNTs resulting in a less flexible and more structural defects [12].

With more than 10 million new cases every year, cancer is one of the most devastating diseases. Though the current treatments of cancer by surgery, radiation, and chemotherapy are successful in several cases; however, these curative methods are likely to kill healthy cells and cause toxicity to the patient [13]. Many patients who succumb to death due to cancer do not die as a result of the primary tumor, but because of the systematic effects of metastases on the other regions away from the original site. One of the aims of cancer therapy is to prevent the metastatic process as early as possible. Therefore, significant amounts of research have been carried out to overcome these problems. The main problem incurred with various chemotherapies for treating cancer is lack of selectivity of the anticancer drug towards cancerous cells. This nonspecificity of the drug limits the therapeutic dose within cancer cells while providing excessive toxicities to normal cells, tissues, and organs and thereby causing several adverse effects. Besides precise tumor targeting and toxicity concerns, drug resistance remains a major obstacle for the treatment of advanced cancerous tumor [14–16]. “Cancer nanotechnology” is the novel emerging field which used nanocarriers like liposome, polymeric nanoparticles, dendrimers, quantum dots, polymersomes, carbon nanotubes, and so forth, for delivering drugs to the target site and thus holds tremendous potential to overcome several problems associated with the conventional therapies [17, 18]. Apart from the several advantages of these nanocarriers, some of them pose challenges of their own. For instance, liposomes have been used as a potential carrier with unique advantages, including protecting drugs from degradation, reduction in toxicity, or side effects, but the applications of liposomes were found to be limited due to the inherent problems such as low encapsulation efficiency, poor solubility of many drugs in the lipid/surfactant solution and rapid leakage of water soluble drug in the presence of blood components as well as unpredictable storage stability [19]. Since precise tumor targeting with reduction in toxicity is the chief objective in cancer therapy, some nanocarriers like immunoliposomes (a class of liposomes) exhibit their inability to actively target the specific cells because the ligands conjugated with liposomes may increase the liposome size and reduce extravasation which may tend to be rapidly cleared by the cells of reticuloendothelial system (RES) [20]. Similarly, targeted nanoparticles (NPs) also faced many challenges. One challenge faced by targeting NPs is that NPs might change the stability, solubility, and pharmacokinetic properties of the carried drugs while another challenge is associated with the shelf life, aggregation, leakage, and toxicity of materials used to make NPs, for example, poly (lactic-co-glycolic acid) (PLGA), a polymer of choice nowadays among the researchers to fabricate NPs because of their low toxicity and high targeting efficiency, but they degrade quickly and do not circulate in tissues long enough for sustained drug/gene delivery which limits their usability in long term cancer therapy. On the other hand, CNTs can persist in the body for weeks, months, or even years, thus limiting their use for repeated treatments [15, 21]. If we talk about dendrimers, they are synthetic, branched macromolecules that form a treelike structure whose synthesis represents a relatively new field in polymer chemistry. Though promising, dendrimers are more expensive than other nanocarriers like CNTs and require many repetitive steps for synthesis, posing a challenge for large-scale production [22]. In the recent years of research, reported data clearly reveals that CNTs have an enormous potential and possess high entrapment efficiency to carry the therapeutic molecule as earliest to the site of cancer cell without activating the immune system and damage to other viable cells although toxicity issues related to CNTs are under research. This review highlights the insights of the CNTs and their potency in delivering the anticancer drugs to combat various metastatic cancer cells specifically.

## 2. Production of CNTs

The production of CNTs complies with the transformation of a carbon source into nanotubes, usually at high temperature and low pressure, wherein the synthesis conditions influence the characteristics of the final product. Since prepared CNTs are usually associated with carbonaceous or metallic impurities, therefore, purification is an essential step to be considered [23]. Methods of CNTs purification include chemical methods, particularly oxidation (gas phase [24], liquid phase [25]), physical methods (filtration [26], centrifugation [27], and high temperature annealing [28], etc.), and multistep purification [29].

**Figure 2:**
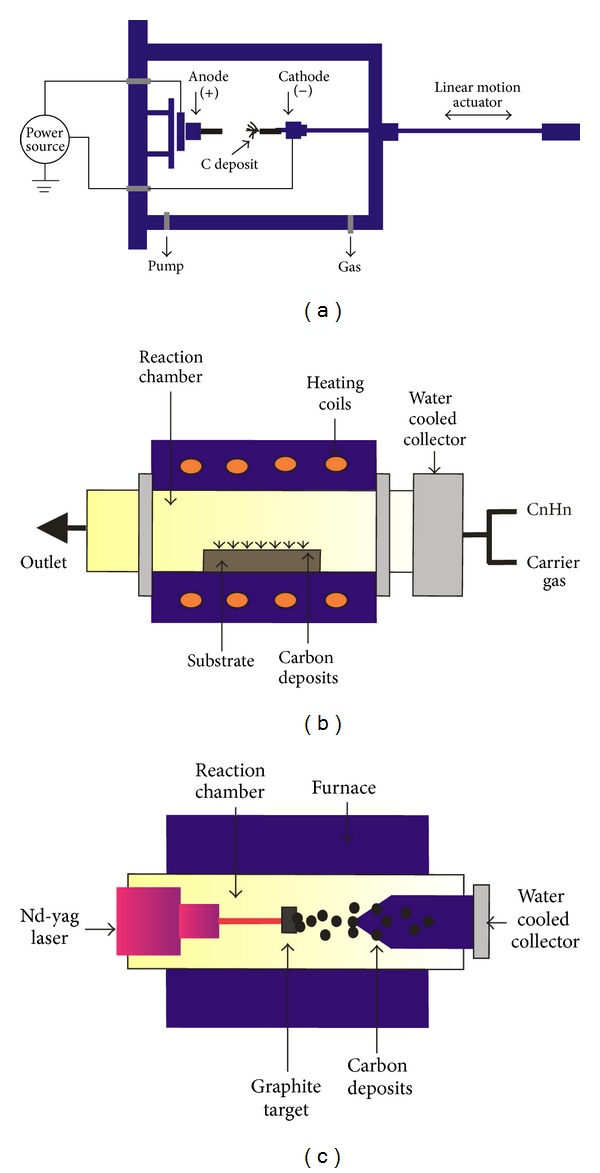
Schematic representation of methods used for carbon nanotube synthesis: (a) Arc discharge method, (b) chemical vapour deposition method, (c) laser ablation method.

**Figure 3:**
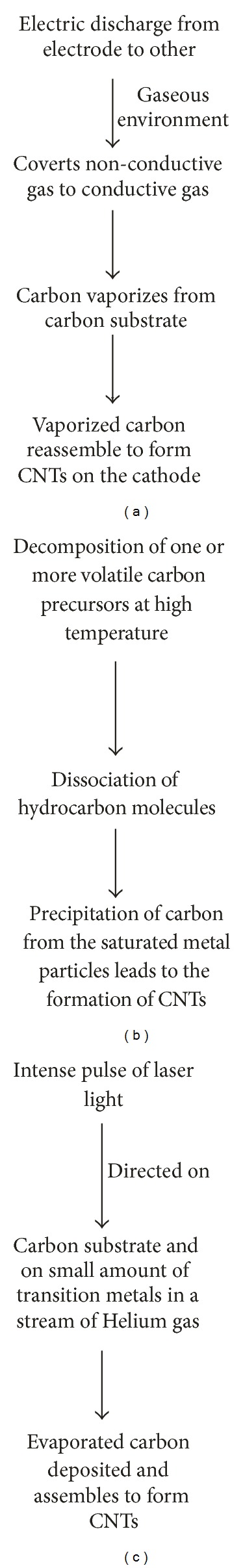
Mechanism of carbon nanotube synthesis: (a) Arc discharge method, (b) chemical vapor deposition method, and (c) laser ablation method.

Presently, there are three main approaches for the synthesis of CNTs (Figures 2 and 3) including electric arc discharge, the laser ablation and the chemical vapor deposition (CVD).

### 2.1. Arc Discharge Method

This method is the oldest method used by Iijima in 1991 when CNTs were first identified [1]. Figures 2(a) and 3(a) showed the synthesis of CNTs by arc discharge method. In this method, an arc is generated, when a DC current of 200A to 20V is applied across two carbon electrodes which are placed in a vacuum chamber that is typically filled with inert gas (Helium, argon) at low pressure (~50~700 mbar). The positive electrode is gradually brought closer to the negative one to strike the electric arc. The electrodes become red hot and such an extreme condition turns the gas into plasma, a fluid of negatively and positively charged particles generating a temperature high enough to vaporize materials. Once the arc stabilizes, the electrodes are kept about a millimetre apart while the CNT deposits on the negative electrode [30, 31]. The two most important parameters to be taken care of in this method are (1) the control of arcing current and (2) the optimal selection of inert gas pressure in the chamber.

Ebbesen and Ajayan [9], for the first time, reported synthesis of CNTs on a large scale and optimized the yield of nanotubes by varying conditions such as type of inert gas, pressure, the nature of current (A.C. or D.C.), the voltage, and the relative rod size. They showed the optimal yield of CNTs at 500 torr pressure. In another study, Ohkohchi et al. studied the growth promoting effect of scandium on nanotubes by using a carbon composite rod containing scandium oxide for the synthesis of CNTs by arc discharge evaporation [32]. Lee et al. and Antisari et al. reported high yield of MWCNTs by electric arc discharge in liquid environments, particularly in liquid nitrogen and deionised water [33, 34]. Alternatively, Wang et al. used sodium chloride solution as a liquid medium because of its significant cooling ability and excellent electrical conductivity than deionised water and concluded successful synthesis of MWCNTs with the formation of a single sheet of SWCNT [35]. Anazawa et al. demonstrated the introduction of magnetic field in arc discharge synthesis to obtain high-purity/defect-free (purity > 95%) MWCNTs [36]. Ando et al. modified this method by a newly developed DC arc plasma jet method for the evaporation of metal doped carbon anode. They showed a high production rate (1.24 g/min) as compared to the conventional method [37]. Cheng et al. devised a hydrogen arc discharge method for the production of SWCNTs with high hydrogen capacity using graphite powder, catalyst metal, and a growth promoter in an atmosphere containing hydrogen [38].

The diameter and the linearity of the SWCNTs can be controlled by the modification in the synthesis procedure. In a study, Farhat et al. altered the inert gas nature by increasing the argon fraction in the inert gas mixture which resulted in the decrease in nanotube diameter [39].

Further, Kajiura et al. also synthesized SWCNTs with narrow diameter distributions using a DC arc discharge method with bimetallic combination (Yttrium-Nickel alloy) as a catalyst and Selenium as parameter [40]. They showed that SWCNTs obtained by this method can be readily purified up to >99% with traditional purification as compared with the above study by Farhat et al.

Among several methods for preparing CNTs, arc discharge is the most practical and inexpensive approach for scientific purposes because the method yields highly graphitized tubes due to high process temperature. However, despite of the above fact, this method produces many byproducts besides CNTs. As a result, the process requires well-controlled purification steps to maintain the quality of nanotubes and eliminate species such as amorphous carbon and metallic nanoparticles [41]. Furthermore, due to the relatively little control over the alignment (i.e., chirality) of the produced nanotubes, the characterization becomes complex.

### 2.2. Chemical Vapour Deposition (CVD)

While the arc discharge method is capable of producing large quantities of unpurified nanotubes, significant effort is being directed towards production processes that offer more controllable routes to the nanotube synthesis. One of the such process is chemical vapour deposition (CVD) that seems to offer the best chance to obtain a controllable process for the selective production of nanotubes with predefined properties [42]. Apart from materials scale-up, controlled synthesis of aligned and ordered CNTs can be achieved by using CVD [43]. The microstructure of the CNT tips synthesized by the CVD technique exhibits well-formed caps compared to other techniques. Therefore CVD is the preferred method for production of CNTs over other methods. The brief schematic representation of this method is given in Figures 2(b) and 3(b). In this method a mixture of hydrocarbon gas (ethylene, methane, or acetylene) and a process gas (ammonia, nitrogen, and hydrogen) is made to react in a reaction chamber on heated metal substrate at a temperature of around 700°C–900°C, at atmospheric pressures. Residual gas diffuses away, whereas free carbon atoms dissolve into the nanoparticles and then segregate to the catalyst surface to form nanotubes [44]. The key parameters include the nature of hydrocarbons, catalysts, and the growth temperature. Depending on the reaction conditions and catalyst preparations, this method may be applied to obtain either SWCNTs or MWCNTs [45].

There are two possible mechanisms for the catalyst assisted nanotube growth, namely, tip growth [46] and root growth mechanism [47]. Balbuena et al. demonstrated the role of catalyst in the growth of SWCNTs by using model Co-Mo catalyst and also studied the role of catalyst/substrate interactions [48]. They found that a strong cluster/substrate interaction increases the cluster melting point, modifying the initial stages of carbon dissolution and precipitation on the cluster surface.

In a study Hoffmann et al. reported the critical effects of NH_3_ or H_2_ on Fe thin film catalyst restructuring which enabled the surface bound growth of SWCNTs at temperature as low as 350°C by CVD [49]. They observed narrow diameter of the SWCNTs formed at low temperature. Various efforts have been taken to modify this technique. One such example is reported by Chen et al. and Choi et al. They showed that by taking advantage of low temperature with the addition of microwave energy that is, microwave plasma-enhanced CVD (PECVD), an increase was seen in the yield of vertically aligned MWCNTs being successfully synthesized [50, 51]. In another study Huisken et al. replaced the global heat source for the furnace with a localized spot heated by a laser and thus studied the suitability of laser assisted CVD (LCVD) for the formation and growth of nanotubes. In this study, they utilized medium power continuous wave CO_2_ laser to irradiate a sensitized mixture of Fe(CO)_5 _ vapour and acetylene to heat the silicon substrate simultaneously on which CNTs were grown [52]. Similarly, Lackey et al. also demonstrated the use of LCVD for the synthesis of CNTs [53]. A novel continuous process registered by Khodadadi et al. involves catalytic chemical vapour deposition (CCVD) of methane on iron floating catalyst in situ deposited on MgO in fluidized bed reactor for the production of CNTs [54].

A major drawback associated with the CVD technique is that there are high defect densities in the MWNT structures grown by this process due to the lack of sufficient thermal energy for annealing CNTs because of relatively low growth temperature [55].

### 2.3. Laser Ablation Method

This method was developed by Smalley et al., in which direct laser beam was focused on transition-metal/graphite composite rods to produce SWCNTs [56]. In the laser ablation process, a pulsed laser is made to strike at a graphite target in a high temperature reactor (furnace) in the presence of inert gas such as helium which vaporizes the graphite target and forms a laser plume. The laser plume contains vaporized carbon and metallic nanoparticles that lead to the reassembling of carbon in the form of carbon nanotubes. The nanotubes develop on the cooler surfaces of the reactor, as the vaporized carbon condenses. Nanotubes produced by laser ablation have higher purity (up to about 90% pure) and their structure is better graphitized than those produced by the arc discharge method. The high cost of laser and small carbon deposit are the major limitations of this method. In addition to this, the method mainly favors the growth of SWCNTs and special reaction conditions are required to generate MWCNTs [57].

Several researchers reported different modifications of the laser ablation method to improve the geometry and yield of SWCNTs (Figures 2(c) and 3(c)). In a study, Maser et al. used continuous wave 250 W CO_2_ laser operating at 10.6 *μ*m wavelength to evaporate graphite/bimetal targets and produced high densities bundles of SWCNTs [58] while Yudasaka et al. showed an improved yield of SWCNTs by pulsed laser over a continuous one [59]. Zhang et al. studied the effect of laser frequency and laser power (or temperature) on the diameter distribution of SWCNTs [60].

Scott et al. demonstrated the growth mechanism for SWCNTs in a laser ablation process. They suggested that in addition to the carbon obtained from direct ablation of the target, other substances like carbon particle suspended in the reaction zone, C_2_ obtained from photodissociation of fullerenes, and other low molecular weight species may also serve as feedstock for nanotube growth [61].

Maser et al. reported that, similar to the arc discharge method, pressure and composition of the inert gas play a significant role in laser ablation synthesis and affect the yield and structural properties of CNTs. Both argon and nitrogen at a pressure between 200 and 400 torr resulted in higher yield of SWCNTs [62]. Apart from this factor another important factor is the carbon source used. Vander Wal et al., in their study, replaced the conventional laser-ablation-furnace approach with the laser ablation in the flame environment. The main advantage of this modification is that it gives an energy efficient approach since a portion of the fuel is burned to create the elevated temperature while the remainder of the fuel and its incomplete combustion byproducts can serve as the reactive carbon source for nanotube synthesis [63].

Thus, both arc discharge method and laser ablation method can be used to produce high quality CNTs. However, efforts to scale up the process are needed to achieve synthesis of larger quantities with maximum purity.

## 3. Functionalization

Despite of the advantages of CNTs in targeting various types of cancer cells, various constraints have been made on the biological and biomedical applications of CNTs due to their lack of solubility in aqueous medium as well as their toxicity caused by the hydrophobic surface. These limitations of CNTs can be overcome by a process called Functionalization [64]. CNT without surface modification, are cytotoxic to certain mammalian cells, for example, pure MWCNTs can injure plasma membrane of human macrophages. Therefore, strategies for surface functionalization including covalent and noncovalent functionalization are carried out on the synthesized CNTs [65]. The process of functionalization is also helpful in conjugating the therapeutic molecule or the ligands to the CNTs either on the surface or on the ends of CNTs to render them active against cancer cells. In this context, recently, a novel immunologically modified nanotube system was invented by Chen using glycated chitosan (GC), a potent immunoadjuvant, as an effective surfactant for single-walled carbon nanotubes (SWCNTs). Upon laser irradiation of target tumor cells, administration of SWCNT-GC resulted in highly effective tumor suppression in animal tumor models, with complete tumor regression and long-term survival in many cases [66]. In a study Dai et al. used oxidised CNT to covalently link fluorescein or biotin (ligand), allowing the formation of biotin-avidin complex with fluorescent streptavidin for observing the penetration pathway of protein-CNT conjugates in the cell [67].

In order to get validated with the modified CNT through functionalization, characterization with respect to pristine CNT is necessary to conduct and to ascertain that the developed CNTs possess altered characteristics with respect to functionalization. There are several analytical techniques which are used in the chemical and structural characterization of modified CNTs [68]. IR spectroscopy (functional group identification) [69, 70], X-ray photoelectron microscopy (degree of functionalization) [70, 71], electron microscopy (structural characterization) [69], Brunauer-Emmett-Teller (BET) analysis (surface area measurement) [72], chemical derivatization (quantification of targeted functional group) [73], thermogravimetric analysis (thermal stability and purity evaluation) [74, 75], and Raman spectroscopy (for ascertaining purity, defects, tube alignment, and structural properties) [76, 77] are some reported techniques used for characterization of CNTs after functionalization.

**Figure 4:**
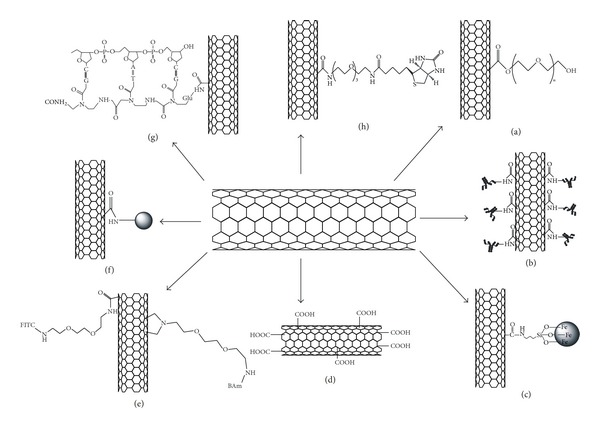
Schematic illustration of functionalization of CNTs with various molecules: (a) Prakash et al. [18], (b) Xiao et al. [78], (c) Xu et al. [70], (d) Gomez-Gualdron et al. [64], (e) Bianco et al. [79], (f) Jiang et al. [80], (g) Williams et al. [81], and (h) Kam and Dai [82].

Schematic representation of functionalized CNTs with various molecules is given in Figure 4. The functionalization can be divided into two main subcategories: noncovalent functionalization and covalent functionalization.

### 3.1. Covalent Functionalization

Covalent functionalization of CNTs with the therapeutically active molecule or the biocompatible surfactants is governed by the oxidation of CNTs using strong acids (conc. H_2_SO_4_ or conc. HNO_3_) which generates substitutable hydrophilic functional groups such as COOH and OH on the CNTs which then further undergo into the chemical reactions such as esterification, amidation, chlorination, bromination, hydrogenation, and Diel's-Alder reaction. In order to get functionalized with these active molecules, CNTs allow side-wall covalent attachment of functional groups by the addition of radicals, nucleophilic carbenes, nitrenes, nucleophilic cyclopropanation, and electrophiles [79, 83, 84]. The method of oxidation results in the opening of the CNT end caps, generating carboxylic groups suitable for enhancing the solubility of the CNTs with improved biocompatibility [85]. It has been shown that a highly negative charge developed as a result of the carboxylation increases the hydrophilicity of CNTs. Covalent linkage of polyethylene glycols increases the hydrophilicity and the solubility of CNTs in aqueous media as well as increasing the size which reduces the rate of clearance of CNTs through the kidneys and tends to increase the circulation time in the plasma. Tour et al. proposed the functionalization of CNTs in acidic media, which yields oxidized CNTs in large and industrial scale quantities [86]. Side wall functionalization of SWCNTs through C–N bond forming substitutions of fluoronanotubes was explored by Khabashesku et al. and reported that this method offers a wide range of further SWCNTs derivatizations including their covalent binding to amino acids, DNA, and polymer matrix [87].

### 3.2. Noncovalent Functionalization

Noncovalent functionalization involves Van der Waals interactions, *π*-*π* interactions, and hydrophobic interactions of biocompatible functional groups with the surface of the CNT. One of the main advantages of this type of bonding is the minimal damage caused to the CNT surface. It has been suggested that noncovalent attachment preserves the aromatic structure and thus the electronic character as compared to pristine CNTs. This type of functionalization can be done by the addition of hydrophilic polymers, biopolymers, and surfactants to the walls of CNTs through weak bonds [88].

A series of anionic, cationic, and nonionic surfactants have been already proposed to disperse nanotubes in aqueous media. Sodium dodecyl sulfate (SDS) and benzylalkonium chloride are other good examples of surfactants that noncovalently aggregated to the nanotube side walls and facilitate the dissolution of CNTs in water. The adhesion between surfactants and nanotube walls becomes very strong due to the *π*-*π* stacking interactions resulted from the attachment of aromatic groups of the amphiphile surfactant in the aromatic network of the nanotube side walls, as evidenced in the case of adhesion of N-succinimidyl-1-pyrenebutanoate [89].

In the solubilization of the CNT, polymers represent a good alternative to surfactants although they do not have a better dispersion efficiency [90]. Amphiphilic polymers or soluble polymers are often used to solubilize CNTs. The main advantage of using polymers instead of small molecular surfactants is that the polymers reduce the entropic penalty of micelle formation. Also, some conjugated polymers have significantly higher energy of interaction with nanotubes than small molecules with nanotubes [91]. In this context, hydrophilic polymer wraps around the tubes and thus modifies the solubility and conductivity properties of the CNTs. For example, polyvinylpyrrolidone (PVP), having polar sides along its long chain, assists the dissolution of PVP/SWCNT aggregates in polar solvents. Similarly Star et al. have substituted poly(metaphenylenevinylene) to suspend SWCNT in organic solvents [92]. Biopolymers can also be used for the functionalization of CNTs. Nucleic acids are certainly ideal candidates to form supramolecular complexes based on *π*-*π* stacking between the aromatic bases and the CNT surface. Zhao et al. reported the DNA adsorption on a single-walled carbon nanotube (SWCNT) in an aqueous environment. The hydrophobic end groups of DNA are attracted to the hydrophobic SWCNT surface of uncharged SWCNTs, while the hydrophilic backbone of DNA does not bind to the uncharged SWCNT [93]. Jiang et al. immobilized biomolecule, bovine serum albumin (BSA) protein via two-step process of diimide-activated amidation on MWCTs. First, carboxylated MWCNTs were activated by N-ethyl-N′-(3-dimethylaminopropyl) carbodiimide hydrochloride (EDAC), forming a stable active ester in the presence of N-hydroxysuccinimide (NHS). Second, the active ester was reacted with the amine groups on the BSA, forming an amide bond between the MWNTs and proteins. This two-step process avoids the intermolecular conjugation of proteins and guarantees the uniform attachment of proteins on carbon nanotubes [80]. William et al. developed a technique to couple SWCNTs covalently to peptide nucleic acid (PNA, an uncharged DNA analogue). Ultrasonically shortened SWCNT ropes were prepared in a 3 : 1 mixture of concentrated H_2_SO_4_ and HNO_3_. Subsequent exposure to 1 M HCl produces abundant carboxyl end groups. This material was then dispersed in dimethyl-formamide (DMF, 99.5%) and incubated for 30 min in 2 mM 1-ethyl-3-(3-dimethyl-aminopropyl)carbodiimide hydrochloride and 5 mM N-hydroxysuccinimide (NHS) to form SWCNT-bearing NHS esters. PNA adducts are formed by reacting this material in DMF for 1 hour with excess PNA [81].

## 4. Mechanism of CNTs Penetration into the Cell

Both types of pure CNTs, the single walled and the multiwalled carbon nanotubes have per se no affinity for cells and also no to cancer cells. That means they have to be functionalized in order to make them able to cross the membrane of the normal cells and even more specifically for targeting them to cancer cells. For this reason, they are basically similar carriers like liposomes, dendrimers, or nanoparticles. However, the advantages of SWCNTs and MWCNTs over other carriers are significant to their hexagonal close-packed cylindrical structure and sp^2^ hybridization which renders them to get easily functionalized with the respective ligand or therapeutic moiety. These functionalized CNTs have an ability to cross cell membranes, but the question arises as to how these functionalized CNTs can recognize their site of action and the route by which they can be delivered to the target cell. Hence, to understand the internalization process, CNTs can be tracked by labeling them with a fluorescent agent (such as fluorescein isothyocyanate) and then monitoring the uptake by using epifluorescence, confocal microscopy, and flow cytometry studies [82, 94]. Additionally, detection of CNTs by nonlabelling methods such as transmission electron microscopy (TEM) or atomic force microscopy has also been conducted by many researchers. Kosuge et al. adopted flow cytometry and confocal microscopy to study the uptake of SWCNTs by the macrophages in murine RAW 264.7 cells. Their observation clearly showed the presence of labelled SWCNTs inside the cells [95]. Transmission electron microscopy was conducted by Bonner et al., on the murine RAW 264.7 cells for the assessment of cellular uptake and sublocalization of MWCNTs. TEM results showed that the RAW 264.7 macrophages successfully engulfed the MWCNTs [96]. Similarly, Sitharaman et al. reported the efficacy of europium (Eu) catalyzed SWCNTs (Eu-SWCNTs), as visible nanoprobes for cellular imaging after observing the internalization of Eu-SWCNTs in the breast cancer cells (SK-BR3 and MCF-7) via cellular endocyte formation as imaged by confocal fluorescence microscopy and TEM [97].

**Figure 5:**
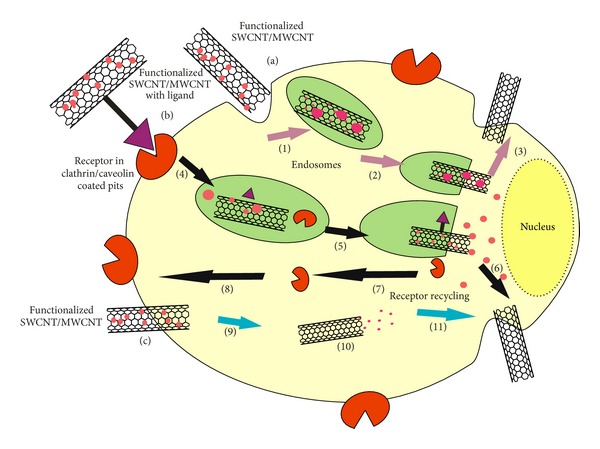
Pathways for the penetration of CNTs into the cell. (a) Nonreceptor mediated endocytosis: (1) membrane that surrounds the drug loaded functionalized CNTs, (2) internalization of drug loaded CNTs, and (3) release of drug; (b) receptor mediated endocytosis: (4) membrane surrounds the CNT-receptor conjugate by forming endosomes followed by internalization, (5) release of drug, and (6, 7, 8) regeneration of receptor; (c) endocytosis independent pathway: (9) direct penetration of drug loaded functionalized CNT and (10) release of the drug.

To conclude about the exact cellular uptake pathway of CNTs is complex and it is believed that there are two possible pathways to cross the cellular membrane (Figure 5) [98]. The first is endocytosis dependent pathway which may be either receptor mediated or nonreceptor mediated and the second is based on endocytosis independent pathway which includes diffusion, membrane fusion, or direct pore transport of the extracellular material into the cell [99]. The process of internalization of CNTs depends on several parameters such as the size, length, nature of functional groups, hydrophobicity, and surface chemistry of CNTs [99, 100].

Endocytosis dependent pathway is an energy and temperature dependent transport process which involves engulfing of extracellular materials within a segment of the cell membrane to form a saccule or a vesicle (hence also called as corpuscular or vesicular transport) which is then pinched off intracellularly into the matrix/cytoplasm of the cell [101]. Furthermore, internalization endocyte formation was shown to be clathrin mediated, caveolin-driven endocytosis, and through macropinocytosis [99]. In case of receptor mediated endocytosis (Figure 5(b)), ligand conjugated-drug loaded CNT binds to the complementary transmembrane receptor proteins and then enters the cell as receptor-ligand complexes in clathrin coated vesicles. After internalization vesicles are formed which were known as early endosomes and due to drop in pH, the ligand dissociates from the receptor. When the receptors are released, the vesicles carrying the extracellular particle fuses with lysosomes and thus trigger the release of the drug particle by the action lysozymes on the endosomes and simultaneously the free receptors thus formed are being recycled to the plasma membrane for conjugating with other ligand conjugated CNTs [102]. An example from the antiangiogenetic area is the targeting of integrin *α*_v_ *β*_3_, which are endothelial cell receptors for extracellular matrix proteins possessing the RGD sequence (arginine-glycine-aspartic acid) and are highly expressed on neovascular endothelial cells. Conjugation of RGD peptides to nanovectors can lead to higher levels of cellular internalization and furthermore affect vascular endothelial growth factor receptor-2 (VEGFR-2) signalling due to intrinsic association with this signalling pathway, leading to downregulation of the receptor and finally to reduced angiogenesis. Another example for active targeting based on ligand-receptor interactions relevant to this area of cancer therapeutics is the interaction of folate with its receptors. Folic acid is a vitamin and necessary for the synthesis of nucleotides, the DNA building blocks. Its counterpart, the folate receptor, is significantly upregulated by a broad spectrum of human cancers, in some cases by two orders of magnitude, facilitating cellular internalization of folate-conjugated nanovectors by receptor-mediated endocytosis. Folate-conjugated nanovectors loaded with anticancer drugs have shown huge potential in overcoming the problem of multidrug resistance by evading P-glycoprotein-mediated efflux, which is considered to be a common problem in cancer drug administration [103].

In a study, Jhao et al. reported the stimulation of Toll-like receptor-9 (TLR9) presented on the intracranial GL261 gliomas bearing mice by CpG oligodeoxynucleotide (CpG) conjugated SWCNTs and concluded that functionalized CNTs were responsible for augmenting CpG prostimulator function by facilitating its uptake through the TLR9 receptor mediated endocyte localization into the glioma cells [104]. Iancu et al. synthesized Human serum albumin (HSA) functionalized MWCNTs inside the malignant liver cells (HepG2 cells) via 60 KDa glycoprotein (Gp60, which is known to function as albumin transcytosis in malignant cells) selective uptake of albumin bounded CNTs by forming an endocyte around it [105]. Similarly fluorescein isothyocyanate labelled lectin conjugated SWCNTs recognises N-acetylgalactosamine containing glycoprotein in MCF-7 breast cancer cell and internalized into the cell as ligand mediated endocytosis [106]. Dhar et al. conjugated cisplatin-platinum (IV) prodrug to amine functionalized SWCNTs through multiple amide linkages and demonstrated its ability to target folic receptors positive (FR+) tumor cells (human choriocarcinoma cells, JAR and human nasopharyngeal carcinoma cells, KB). Results obtained from the fluorescence microscopy analysis clearly stated the applicability of the conjugated system to selectively target FR+ receptors and the internalization of the system was through the folic acid receptor mediated endocytosis [107].

In general, the long CNTs (>1 *μ*m in length) were taken up by the process of phagocytosis (a part of endocytosis) which was mainly conducted by the macrophages, monocytes, and neutrophils, while the shorter CNTs (length from a few to several hundred nanometers) were mainly internalized by pinocytosis [108]. It was found that altering the hydrophobicity of the CNTs by conjugating them with phospholipids significantly alters the uptake of CNTs by the cells as observed by Kapralov et al. They compared the internalization of the surfactant (phosphatidylcholines and phosphatidylglycerols) conjugated SWCNT with pristine SWCNT in the murine RAW 264.7 cells and the data obtained from flow cytometric analysis clearly states that the adsorbed phospholipids significantly enhanced the uptake of SWCNTs via phagocytosis as phospholipids are known to greatly associate with the phospholipids head group of the cellular membrane in comparison to pristine or uncoated SWCNT [109].

In case of nonreceptor mediated endocytosis (Figure 5(a)), a small portion of the plasma membrane surrounds the drug loaded CNTs and then pinches off intracellularly as an endocyte vesicle. The process is governed by the intrinsic property of the CNTs as well as functional groups present on it. The endosomes thus formed are eventually converted into the lysosomes and ultimately result in the drug release [102]. Liu et al. developed SWCNT conjugated with paclitaxel (PTX) and reported the nonreceptor mediated endocytosis mechanism for the cellular uptake in murine 4T1 breast carcinoma cells [110].

Islam et al. investigated the cellular uptake of pluronic copolymer-stabilized, purified ~145 nm long single walled carbon nanotubes (SWCNTs) through a series of complementary cellular, cell-mimetic, and in vitro model membrane experiments. The Raman intensity distribution, obtained from the G-band (1590 cm^−1^), shows SWCNT concentration localized to the midplane of a fixed, hematoxylin-labelled cell. SWCNTs were preferentially located within cells versus the extracellular regions, and most SWCNTs were localized in the perinuclear region. SWCNTs localized within fluorescently labelled endosomes and confocal Raman spectroscopy showed a dramatic reduction in SWCNT uptake into the hematoxylin-labeled HeLa cell at 4°C compared with 37°C, after being incubated for 2 days. These data suggested energy-dependent endocytosis. To confirm this, a direct measurement to check the endocytosis was conducted in which endocytosis in HeLa cells was visualized using a Green Fluorescent Protein, GFP-tagged RhoB-GTPase, which labels endosomes in mammalian cells. On confocal imaging it was confirmed that the internalization of SWCNTs was occurring through the endocyte formation as there was a slight increase in endosome numbers per cell with increased time of exposure to SWCNTs, with a statistically significant increase in endosome number at 20 and 25 minutes [111].

In endocytosis independent pathway there is a direct translocation of CNT through the plasma membrane into the cytoplasm which has been termed by some researchers as the “nanoneedle” mechanism [100]. This pathway includes processes such as diffusion, membrane fusion, and direct pore transport (Figure 5(c)). Individually dispersed CNTs in aqueous solutions have been experimentally demonstrated to be able to enter the cytoplasm of cells by directly crossing the cell membrane. Such cellular uptake of CNT, which is not influenced by the presence of endocytosis inhibitor (such as sodium azide), suggests the endocytosis independent pathway of internalization [112]. The mechanism of how CNT enters cells via the insertion and diffusion is poorly understood. Some theoretical studies suggest a two-step process in which, first, the tubes accommodate onto the lipid cell membrane and then orient to adopt the transmembrane configuration. In this model, the internalization of nanotubes into the cells was spontaneous and was mediated by the lipid membrane and that the hydrophilic interactions and/or static charge interactions between the tubes and the lipid membrane which drove the translocation of the nanotubes [113]. The conformation of CNT perpendicular to the plasma membrane during uptake suggested a mechanism similar to nanoneedles, which perforate and diffuse through the lipid bilayer of the plasma membrane without inducing cell death [79]. Rawson et al. reported the first demonstration of a direct interface of vertically aligned SWCNTs (VASWCNTs) with eukaryotic RAW 264.7 mouse macrophage cell line. VASWCNTs entered the cells naturally due to its needle-like structure without application of any external force owing to endocytosis independent pathway for internalization [114].

## 5. Application of CNTs in Cancer Treatment

For decades, human immortal cancer cell lines have constituted an accessible, easily usable set of biological models with which to investigate cancer biology and to explore the potential efficacy of anticancer drugs is of less tedious work. Currently, various ex vivo studies, such as cell line studies, cellular uptake studies, fluorescent microscopy, and flow cytometry, are carried out for this purpose.

Various cancer cell lines were cultured with modified CNTs (functionalization on the surface and ends of the CNTs, and by conjugating CNTs with ligands) and evaluated for therapeutic efficacy, cell viability, cell survival assays, and cell apoptosis. Ex vivo studies specifically used in the evaluation of CNTs for cancer chemotherapy are shown in Table 1.

### 5.1. Brain Cancer

Brain cancer is the leading cause of cancer-related death in the US in patients under the age of 35. Anaplastic astrocytomas (Grade III) and glioblastomas (Grade IV) are most aggressive brain cancers with survival period of 24 and 9 months, respectively [138]. Children who survive their brain cancers (mainly medulloblastomas) often suffer substantial adverse effects related to the toxicities of therapy on the developing nervous system [139]. Currently available systemic chemotherapy is less effective due to presence of the blood-brain barrier (BBB) which restricts the penetration of most drugs into the brain. Recently, a number of CNT-based targeting approaches have been developed for the treatment of brain cancer and a brief account is presented below.

Vittorio et al. investigated the biocompatibility of MWCNTs with cultured Human neuroblastoma cells SH-SY5Y. Reactive oxygen species (ROS) are chemically reactive molecules containing oxygen. ROS can damage cellular proteins, lipids, and DNA leading to fatal lesions in cells that contribute to carcinogenesis. In vitro experiments showed loss of cell viability was minimal with no intracellular ROS detected with prolonged cultures and continued propagation in the presence of 99%, 97% pure MWCNTs and acid-treated 97% pure MWCNTs but no significant decrease in the proliferation of cells incubated for 3 days was observed with the cells cultured with 99% pure MWCNTs. After conducting WST-1 assay it was assessed that, on increasing the concentration of MWCNTs from 5 *μ*g/mL to 500 *μ*g/mL, purity and surface oxidation of MWCNTs seem to downregulate the % cell viability and at 5 *μ*g/mL 100% cells are viable. Hence it was concluded that the concentration of 5–10 *μ*g/mL seems ideal for gene and drug therapy against cancer [115].

Xing et al. synthesized phospholipid-bearing polyethylene glycol (PL-PEG) functionalized SWCNTs conjugated with protein A which was further coupled with the fluorescein-labeled integrin *α*_v_ *β*_3_ monoclonal antibody to form SWCNT-integrin *α*_v_ *β*_3_ monoclonal antibody (SWCNT-PEG-mAb). Confocal microscopy revealed that SWNT-PEG-mAb showed a much higher fluorescence signal on integrin *α*_v_ *β*_3_-positive U87MG cells and presented a high targeting efficiency with low cellular toxicity, while, for integrin *α*_v_ *β*_3_-negative MCF-7 cells, no obvious fluorescence was observed which clearly reveals low targeting efficiency of the functionalized SWCNTs, demonstrating that the specific targeting of integrin *α*_v_ *β*_3_-positive U87MG cells was caused by the specific recognition of integrin *α*_v_ *β*_3_ on the cellular membrane by the *α*_v_ *β*_3_ monoclonal antibody [116].

Oxidized MWCNTs cannot only be distributed in the brain but may accumulate in tumors after conjugating with specific ligands and also possess an ultrahigh surface area for efficient loading of anticancer drug. Ren et al. developed a dual targeting PEGylated MWCNTs and loaded with a targeting ligand angiopep2 (ANG) and doxorubicin, respectively, to target low density lipoprotein receptor-related protein receptor which is overexpressed on the blood brain barrier (BBB) and C6 glioma cells. In vitro intracellular tracking and in vivo fluorescence imaging demonstrated the ideal dual targeting of the developed system which was attained by the higher transcytosis capacity and parenchymal accumulation by the angiopep-2 and can be considered a material of choice to cross blood brain barrier as well as to specifically recognise their lipoprotein receptors present on the glioma cells for directing the site specific release of anticancer drug. C6 cytotoxicity, hematology analysis, and CD68 immunohistochemical analysis reveals better antiglioma effect with good biocompatibility and low toxicity of O-MWCNTs-PEG-ANG, compared with that of free doxorubicin [117].

The existence of cancer stem cells (CSCs) or stem-like cancer cells (SLCCs) is regarded as the cause of tumor formation and their recurrence. However, the origin of such cells remains controversial with two competing hypotheses: CSCs are either transformed tissue adult stem cells or dedifferentiated from transformed progenitor cells [140]. Wang et al. explored the potential of CD133 monoclonal antibody (anti-CD133) conjugated SWCNTs for therapeutic targeting of CD133 CSCs. Glioblastoma (GBM)-CD133^+^ cells were selectively targeted and eradicated whereas GBM-CD133^−^ cells remained viable. Furthermore, anti-CD133-SWCNTs pretreated GBM-CD133^+^ cells were irradiated with near-infrared laser for 2 days and showed no sign of sustainability of CSCs for tumor growth after xenotransplantation in nude mice. From this report it is stated that monoclonal antibody conjugated SWCNTs are capable of selectively targeting the CSCs as well as blocking their recurrence [119].

### 5.2. Blood Cancer

Leukemia is a cancer that begins in the bone marrow (the soft inner part of some bones), but in most cases, moves into the blood. It can then spread to other parts of the body, such as organs and tissues. Acute lymphoblastic leukemia (ALL), one of the four main types of leukemia, is a slow-growing blood cancer that starts in bone marrow cells called lymphocytes or white blood cells. Once these white blood cells are affected by leukemia, they do not go through their normal process of maturing. The lymphocytes continue to reproduce and build up and invade the blood fairly quickly. ALL is an aggressive type of leukemia; without treatment, most patients with acute leukemia would live only a few months [141].

An enhanced targeted delivery of daunorubicin (Dau) to acute lymphoblastic leukemia was achieved by Taghdisi et al., they developed a tertiary complex of Sgc8c aptamer, daunorubicin, and SWCNT named as Dau-aptamer SWCNTs. Flow cytometric analysis showed that the tertiary complex was internalized effectively into human T cell leukemia cell (MOLT-4 cells) but not to U266 myeloma cells [121].

### 5.3. Breast Cancer

Breast cancer (BC) has become the most common malignancy and the leading cause of cancer-specific death in women, according to GLOBOCAN 2008 estimates [142]. Overexpression of human epidermal growth factor receptor 2 (HER2), also known as c-erbB-2 or HER2/neu, is approximately 20%–25% responsible for invasive BC. With an increasing understanding of the role of HER2 in tumor proliferation, angiogenesis, and metastasis, novel special treatment strategies for this HER2-positive subtype of BC have been validated and are increasingly used in clinical practice. One of the most important treatment strategies is to block the signal pathway of HER2/neu; this is defined as targeted therapy [143].

In a study, Pan et al., investigated the efficiency of MWCNTs to deliver gene to the tumor cell for cancer therapy. In this work, they fabricated MWCNTs modified with polyamidoamine dendrimer which were further conjugated with FITC-labelled antisense c-myc oligonucleotides (asODN). Human breast cancer cell line MCF-7 cells and MDA-MB-435 cells were incubated with modified MWCNTs (asODN-dMNTs). Fluorescence developed by the FITC revealed the cellular uptake of asODN-dMNTs within 15 min. These composites inhibit the cell growth in time and dose dependent means and downregulated the expression of c-myc gene (overexpression of this gene amplify the expression of HER2) and C-Myc protein [123].

A chemically functionalized SWCNT carrier has been developed for the effective delivery of SiRNA and SiRNA-MDM2 complexes to the breast carcinoma B-Cap-37 cells. Results proved the high efficiency of F-SWCNT in carrying SiRNA to the carcinoma cells and the new F-SWCNT-SiRNA-MDM2 complexes caused 44.53% inhibition of proliferating B-Cap-37 carcinoma cells for 72 hours by downregulating the expression of c-myc gene [125].

Panchapakesan et al. discovered the explosive nature of SWCNT which can act as a potent therapeutic nanobomb agents for killing breast cancer cells. In his work, he adsorbed water molecule on the SWCNT, which upon exposure to laser light of 800 nm at light intensities of approximately 50–200 MW/cm^2^ which is sufficient to transform optical energy to thermal energy and cause the evaporation of water molecules which built extreme pressure in SWCNT causing them to explode in the suspension of human BT-474 breast cancer cells in phosphate buffered saline solution and render the cells to death. The presence of bubbles around the dead cells revealed the boiling effect caused by SWCNT explosions [126].

A water soluble SWCNT-Paclitaxel (PTX) conjugate has been developed by conjugating PTX to functionalized polyethylene glycol SWCNTs via a cleavable ester bond. SWCNT-PTX has been found to be highly efficient in suppressing tumor growth when compared with clinical taxol in a murine 4T1 breast cancer cells, which has been attributed to the extended blood circulation (due to PEGylation) and tenfold higher tumor PTX uptake by SWCNT delivery, probably through enhanced permeability and retention (EPR) effect [110].

### 5.4. Colon Cancer

Colorectal cancer is the leading cause of death amongst the men and women worldwide and afflicts more than 135,000 patients per year in America. This cancer has usually been viewed as a homogeneous entity rather than a complex heterogeneous disease developing through multiple genetic and epigenetic abnormalities, such as defective DNA mismatch repair (dMMR) and the CpG island methylator phenotype (CIMP) [144].

Abdolahad et al. utilize the vertical arrays of MWCNTs for entrapping the metastatic human colon adenocarcinoma SW-48 cells and HT-29 cancerous cells. Due to the extreme deformability and softness of higher metastatic malignant cells, they exhibit higher fraction of entrapment by the vertically aligned MWCNTs as compared to the less deformable and rigid lower grades of metastatic cancerous cells. This new application of MWCNTs distinguishes the healthy and highly deformable cancerous cells more precisely than SWCNTs and also showed better delivery of anticancer drugs to these cancer cells [127].

Triple functionalized SWCNTs were fabricated with an anticancer drug (Doxorubicin), a monoclonal antibody and a fluorescent marker (fluorescein) at the noncompetitive binding sites on the SWCNTs for targeting the cancer cells. Confocal laser microscopy reveals the bovine serum albumin-antibody specific receptor mediated uptake of SWCNTs by the human colon adenocarcinoma cell WiDr cells with subsequent targeting of doxorubicin intracellularly to the nucleus [128].

### 5.5. Liver Cancer

Hepatocellular carcinoma (HCC) is a highly prevalent malignancy, especially in Asia. Liver cirrhosis is the strongest predisposing factor for HCC, accounting for approximately 80% of patients with this disease. In the United States, Europe, and Japan, hepatitis C virus (HCV) infection is the major etiology of liver cirrhosis and HCC. Hepatitis virus B (HBV) infection, however, is the leading cause of HCC development in most Asian countries other than Japan. In addition to HBV and HCV infection, alcoholic cirrhosis and metabolic disorders can also act as risk factors for HCC [145]. c-Myc is among the most frequently overexpressed genes in human cancers. Overexpression of c-Myc in hepatic cells leads to the development of hepatocellular carcinoma [146]. However, an attempt has been made by Pan et al. to suppress the expression of c-myc gene and C-Myc protein in the tumor bearing cell.

Polyamidoamine dendrimer modified CNTs (dMWCNTs) were fabricated for the efficient delivery of antisense c-myc oligonucleotide (asODN) into liver cancer cell line HepG2 cells. asODN-dMWCNTs composites were incubated with HepG2 cells and confirmed to enter into tumor cells within 15 min by laser confocal microscopy. These composites inhibited the cell growth in time and dose dependent means and downregulated the expression of the c-myc gene and C-Myc protein. These composites exhibit maximal transfection efficiencies and inhibition effects on tumor cells when compared to CNT-NH_2_-asODN and dendrimer (asODN) alone [123].

Meng et al. constructed a highly effective targeted DDS based on chitosan and folic acid modified SWCNTs for controllable loading/release of anticancer agent doxorubicin (DOX). The obtained DDS not only effectively killed the hepatocellular carcinoma SMMC-7721 cell lines and depressed the growth of liver cancer but also displayed much less in vivo toxicity than free doxorubicin [129].

### 5.6. Lymph Node Metastasis

The presence of lymph node invasion is one of the strongest indicators for prognoses of distant metastasis and survival in most cancers. In the multistep process of cancer metastasis development, invasion into a vascular or a lymphatic system has generally been believed to be a key step of tumor cell dissemination. Once tumor cells acquire abilities of intravasation and survival in an unfavorable vascular environment, they circulate around the whole body parts to form new tumors at the secondary site [147]. Lymph node metastasis is a powerful predictor of recurrence and death in patients with cutaneous melanoma. Metastasis to regional lymph nodes develops during the course of the disease in approximately 30% of patients with cutaneous melanoma [148].

Yang et al. compared the in vitro and in vivo potential therapeutic effect of gemcitabine (GEM) loaded magnetic MWCNTs (mMWCNTs) with that of gemcitabine loaded magnetic-carbon particles (mACs). His finding reflects the high antitumor activity in human pancreatic cancer BxPC-3 cells of both the systems when compared along with free drug. Due to superparamagnetic behaviour of mMWCNTs-GEM, their magnetic moments tend to align along the applied field leading to net magnetization which greatly affects the interaction of mMWCNTs-GEM with the cellular membrane and thus they were found to be superior than mACs-GEM in successful inhibition of lymph node metastasis after following subcutaneous administration under the impact of magnetic field [130].

### 5.7. Kidney Cancer

Renal cell carcinoma (RCC) is responsible for approximately 80% of primary renal cancers, and urothelial cell carcinoma (UCC) accounts for the majority of the remainder (20%). The most common histological subtype of RCC is the conventional or clear cell (ccRCC). The occurrence of ccRCC is due to the defunctioning of the Von Hippel-Lindau (VHL) tumor suppressor gene (TSG), located on chromosome 3p. Loss of functioning of the VHL protein leads to stabilization of hypoxia-inducible factors and nuclear transcription factors that in turn can activate the transcription of many genes including those encoding vascular endothelial growth factor (VEGF) and platelet derived growth factor [149]. RCC is a highly aggressive tumor and also the most lethal of urologic malignancies with an estimated 88,400 new cases and 39,300 kidney cancer-related deaths from RCC in Europe [150].

Cui et al. investigated the interaction between SWCNTs and human embryo kidney HEK-293 cells intended to explore SWCNT biocompatibility and safety. It was found that SWCNTs can inhibit the proliferation of HEK-293 cells, induce the cell apoptosis, and decrease cell adhesive ability in a time and dose dependent manner. SWCNTs induce changes in the cell cycle which could be attributed to the decrease in the number of cells in the S-phase due to upregulated expression of P16 which inhibits the cyclin dependent kinase activity of CdK2, CdK4, and CdKr and therefore prevents the cells from entering an S-phase and subsequently arresting the cell cycle in the G1 phase [131].

### 5.8. Cervical Cancer

Oncogenic human papillomavirus (HPV) has a causal role in nearly all cervical cancers and in many vulvar, vaginal, penile, and oropharyngeal cancers. HPV types 16 and 18 are majorly responsible for 70% of cervical cancers [151]. In HPV-associated cancers, oncogenic antigens E6 and E7 were overexpressed on the tumor cells and thus, they represent an ideal target for developing antigen-specific immunotherapy for the control of cervical cancer [152].

Wu et al. developed a novel approach of utilizing natural biocompatible polymer chitosan for imaging the tumor cells. In this assay, SWCNTs were modified by chitosan (CHIT) fluorescein isothyocyanate (FITC). This was further conjugated with folic acid (FA), as mostly cancers cells overexpress folic acid receptors, to construct the functional FITC-CHIT-SWCNT-FA conjugate. These novel functionalized SWCNTs were found to be soluble and stable in phosphate buffer saline and can be readily transported inside the human cervical carcinoma HeLa cells. Combining the intrinsic properties of CNTs, versatility of chitosan, and folic acid, FITC-CHIT-SWCNT-FA can be used as potential devices for targeting the drug into the tumor cells and also for imaging them [134].

Five types of CNTs suspensions were prepared by Zhang et al., by dispersing SWCNTs, acid-treated SWCNTs, MWCNTs, acid treated MWCNTs, and amylose wrapped SWCNTs, individually in water, and the influence of these scaffolds on human cervical carcinoma HeLa cells was investigated by WST-1 assay, acridine orange/ethidium bromide double staining, and 1,1′-dioctadecyl-3,3,3′,3′ tetramethylindocarbocyanine perchlorate staining. The results indicated that both “dot like” and “dash like” focal adhesion kinases (FAKs) were mainly distributed at the periphery of the cells cultured on SWCNTs and on acid-treated SWCNTs and due to this they were found undergoing apoptosis with damaged cell membrane and condensed chromatin; however, cells cultured on MWCNTs, acid-treated MWCNTs, and amylose wrapped SWCNTs were found to be viable which is due to the distribution of “dot like” focal adhesion kinases (FAKs) in the whole cell body of the cells [135].

### 5.9. Prostate Cancer

Prostate cancer is a slow growing cancer and early propagation of cancer cells occurs before the disease become clinical. Cases of prostate cancer in USA estimates 238,590 in the year 2013 out of which 29,720 cases of death due to prostate cancer have been reported in SEER stat facts sheet published by National Cancer Institute, USA [153]. Prostate cancer antigen 3 (PCA3) has been validated as the principal molecule associated with prostate cancer (PCa). The PCA3 gene is located on the chromosome 9q21–22 and was molecularly characterized as the prostate cancer-specific gene, highly overexpressed in almost all prostate tumor specimens and PCa metastasis. Here we discuss a study using human prostate cancer cell line with respect to CNTs [154].

Li et al. developed a novel targeting SiRNA delivery system by using SWCNTs which was chemically functionalized with polyethylenimine and bound by DSPE-PEG 2000 maleimide for further conjugation with tumor targeting Asn-Gly-Arg (NGR) peptide. This novel system efficiently crosses human prostate cancer cell PC-3 cell membrane in vitro and induces more severe apoptosis and suppression in the proliferating cells. The combination of near-infrared photothermal therapy and RNAi significantly enhanced the antitumor activity without causing toxicity to other organs [137].

## 6. Toxicological Assessment of CNTs

Despite of the exciting prospects of CNTs in drug delivery, there are some factors which limit the applications of CNTs. Presence of impurities, nonuniformity in morphology and structure, large surface area (leads to protein opsonization), hydrophobicity, insolubility, and tendency of CNTs to bundle together are some obstacles for their nanomedical applications [23, 155].

Another key obstacle is the toxicity of CNTs. Entrance into the body is physical, and usually few nanoparticles enter the body; however, once there, they are persistent due to their limited metabolisms, so their removal is slow, and chronic cumulative health effects are studied. The underlying mechanisms of CNT toxicity include oxidative stress, production of cytokines, chemokines and inflammatory responses, malignant transformation, DNA damage and mutation (errors in chromosome number as well as disruption of the mitotic spindle), the formation of granulomas, and interstitial fibrosis [156, 157].

In view of carcinogenicity of CNTs, SWCNTs were directly instilled into the lungs of the animals, it was found that exposure to SWCNTs at a high concentration leads to the development of granulomas in rodents and a concentration of 0.5 mg/m^3^ and 2.5 mg/m^3^ for MWCNTs induces microgranulomas with the inflammation in the alveoli [158–160]. Similarly, in a study by Kanno et al., demonstrated the carcinogenic potential of MWCNT to induce multiple mesothelioma with severe peritoneal adhesion when administered intraperitoneally to p53 heterozygous mice. This may be due to its structural similarities (size/shape) to asbestos as well as persistency in the organism, while in an another study reported by Takanashi et al., and it was stated that subcutaneously implanting the MWCNTs in to the rasH2 mice did not develop neoplasm [161–163].

In view to the inflammatory responses with CNTs, Monteiro-Riviere et al. exposed human epidermal keratinocytes (HEK) to MWCNTs and found that MWCNT induces the release of proinflammatory cytokine interleukin 8 from HEK which indicates the irritation response on target epithelial cells [164]. Similarly, upon subcutaneously administering MWCNT at 0.1 mg/kg and 1 mg/kg showed acute inflammation characterized by vasodilatation, edema formation, neutrophil infiltrate, tissue damage and also elicited hyperalgesic response (as seen by the increase paw withdrawal of animal) [165]. In a study, Pons et al. investigated the immunomodulatory activity of MWCNTs in peripheral blood mononuclear cells (PBMCs) from healthy donors and mite-allergic subjects. It was observed that MWCNTs may either promote or suppress immune responses with the type of Toll-like receptor agonist the cells are stimulated with. Basal secretion of all TNF-*α*, IL-2, IL-5, IL-6, IL-12/23p40, or IFN-*γ* was not altered by MWCNTs in PBMCs derived from both healthy donors and allergic subjects but significantly increased in the release of TNF-*α*, IL-6, and IL-12/23p40 was observed in PBMCs stimulating the Toll-like receptor (TLR2, TLR3, and TLR4) agonist [166].

Among the many toxicity pathways, interference with cytoskeleton and fibrous mechanisms, cell signalling, and membrane perturbations are some of the effects resulting from exposure to CNTs [157]. In a study, exposure of MWCNT to the coculture of SAEC (small airway epithelial cells), a Type II epithelial cell, and HMVEC (human microvascular endothelial cells), increases the VEGFA (vascular endothelial growth factor), soluble adhesion molecules (sICAM-1 and sVCAM-1) protein levels, as well as increases the intracellular phospho-NF-*κ*B, phospho-Stat3, and phospho-p38 MAPK (mitogen-activated protein kinases) which tends to induce multiple changes in the endothelial cell barrier, including an increase in reactive oxygen species, actin rearrangement, loss of VE-cadherin (vascular endothelial) at the cell surface, and an increase in endothelial angiogenic ability [167].

It was reported that length of MWCNTs was found to exert effects on the biomembranes; when the distribution of MWCNTs (3–14 *μ*m length) in RAW264 cells was observed under a light microscope, MWCNTs were located on the surface of the plasma membrane and a portion of them seemed to be stucked on it which tends to increase the permeability defects of the plasma membrane lipid bilayer while shorter (1.5 *μ*m) MWCNTs were significantly less toxic [168, 169]. In a study, interference of CNTs with cytoskeleton was investigated by Shvedova et al., and exposure of cultured human epidermal keratinocytes (HaCaT) to SWCNTs induces oxidative stress and results into loss of cell viability, indicating that dermal exposure to CNTs may lead to these altered skin conditions [170].

Not only bare CNTs showed toxicity, but also functionalized CNTs were also reported to cause toxicities; as in a study by Tian et al., covalently functionalized MWCNTs with carboxylate (COOH), polyethylene glycol (PEG), amine (NH2), side-wall amine (sw-NH_2_), and polyetherimide (PEI), respectively, were screened for toxicity in bronchial epithelial cells and BEAS-2B and TPH-1 cells. It was observed that anionic functionalization (COOH and PEG) decreased the production of profibrogenic cytokines and growth factors (including IL-1B, Transforming growth factor beta 1 (TGF-B1) and platelet derived growth factor-AA (PDGF-AA)), while neutral and weak cationic functionalization (NH_2_ and sw-NH_2_) showed intermediary effects. In contrast, the strongly cationic PEI functionalized MWCNTs induced biological effects. Compared to pristine MWCNTs, strong cationic PEI-MWCTs induced significant lung fibrosis, while carboxylation significantly decreased the extent of pulmonary fibrosis [171]. But the toxicity of f-MWCNTs at varying degrees of carboxylation was assessed by Jain et al., in a murine macrophage RAW 264.7 cell line, a model for liver Kupffer cells. Increased in vitro cytotoxicity was found to be directly proportional to carboxylation density which was associated with a concurrent increase in the number of apoptotic cells and production of reactive nitrogen species (RNS) and reactive oxygen species (ROS) [172].

Acid-functionalized SWCNTs induce adverse effects in murine peritoneal macrophages which were related to the conversion of microtubule-associated protein light chain 3, LC3-I to LC3-II, and the accumulation of SWCNT in macrophage lysosomes, leading to lysosome membrane destabilization, which indicates reduced autophagic degradation [173]. Campagnio et al. studied the toxicity of PEGylated SWCNTs in pregnant mice. A dose of 10 *μ*g/mouse has no adverse effects both on embryos and dams but at a dose of 30 *μ*g/mouse, and teratogenic effects associated with placental damage were detected both in embryos and dams (dose given as a single bolus or as multiple doses). Hepatic damage in dams was seen only in the multiple exposure groups. PEGylated SWCNTs reached the conceptus when administered early in pregnancy and at later stages it was detected in the placenta and the yolk sac but not in embryo [174].

Before the widespread utilization of CNTs in the medical science, it is important to note that the chronic toxicity of CNT must be experimentally studied and the appropriate safeguards must be taken against the possible interactions among the CNTs and biological systems.

## 7. Conclusion

CNT represents a novel class of carriers for the delivery of drugs in a site specific and target oriented manner. CNTs possess extraordinary physical, chemical, and mechanical properties, which make them as a potent biological carrier to deliver anticancer drugs. Studies have clearly shown that functionalization of CNT and further derivatization with biodegradable polymers render them compatible with biological systems. Due to their unique chemistry, hexagonal arrangement of carbon atoms, various sites are available for both covalent and noncovalent functionalization with the therapeutically active molecule or protein macromolecules which envisaged the potential of CNT as nanocarrier for the site specific delivery of therapeutic agent including peptides, proteins, nucleic acid, and other small drug molecules for targeting various cancer cells. These functionalized CNTs possess high propensity to traverse cell membrane either via endocytosis dependent or independent pathways.

Thorough investigations have been performed in this review on various synthesis and modification routes for the production of purified CNTs and their role in combating cancer. Various ex vivo models based on different cancer cell lines were studied to determine the pharmacokinetic and pharmacodynamic parameters of anticancer compounds, that is, being carried by the biocompatible nanosized carbon tubes at the targeted site on cancer cells. All the observations and results cited in this review evidently endorse the usefulness of functionalized CNTs as a potential carrier for the anticancer molecule to target the cancer cell without causing toxicity to other viable cells. Also, the usefulness of cell lines has greatly validated the results for the assessment of in vivo therapeutic and diagnostic efficacy for cancer treatment and reduces the dependency on animal and human models for the treatment of cancer at the preclinical and clinical study trial level. This compilation of the literature provides useful information to researchers for exploring the further scope of CNTs in the medical science.

## Figures and Tables

**Table 1:** Impact of functionalized CNTs on cancer cell lines.

Cancer type	CNT properties		Bioactive molecule	Objective	Cell line	Repercussion of the study	Reference
	Type	Functionalization					
Brain cancer	MWCNTs	—	—	Influence of purity and surface oxidation on cytotoxicity of MWCNTs with human neuroblastoma cells	Human neuroblastoma cell line SH-SY5Y	With prolonged cultures, loss of cell viability was minimal for preparations with 99% purity, but significant adverse effects were detected with 97% purity and with acid treated preparations. A concentration of 5–10 *µ*g/mL of MWCNTs seems ideal for gene and drug therapy against cancer	[115]
	SWCNTs	Phospholipid-polyethylene glycol (PEG), protein A, fluorescein labeled integrin *α*_v_ *β*_3_ mAb	—	Functional SWCNTs based on an integrin *α*_v_ *β*_3_ mAb for the highly efficient cancer line	Human U-87MG glioblastoma cell, MCF-7	In vitro study revealed that SWCNT-PEG-mAb presented a high targeting efficiency on integrin *α*_v_ *β*_3_-positive U87MG cells with low cellular toxicity.	[116]
	MWCNTs	Angiopep-2, PEGylated	Doxorubicin	Targeted delivery of anticancer drugs to brain glioma by PEGylated oxidized MWCNTs modified with angiopep-2.	C6 glioma cells	Angiopep-2 modified PEGylated MWCNTs showed better antiglioma activity, good compatibility, and low toxicity.	[117]
	MWCNTs	Poly(acrylic acid) and folic acid	Doxorubicin	Dual targeted delivery of doxorubicin to cancer cells using folate conjugated magnetic MWCNTs.	U87 human glioblastoma cells	Dual targeting of doxorubicin from magnetic MWCNTs showed enhanced cytotoxicity toward U87 human glioblastoma cells compared with free doxorubicin.	[118]
	SWCNTs	CD133 monoclonal antibody (mAB)	—	Photothermolysis of glioblastoma stem like cells targeted by CNT conjugated with CD133 mAB.	GBM-CD133^+^ and GBM-CD133^−^ cells	GBM-CD133^+ ^cells were selectively targeted and eradicated whereas CD133^−^ cells remained viable after incubation with SWCNT-CD133mAB. GBM-CD133^+ ^cells pretreated with SWCNT-CD133mAB and irradiated by near-infrared laser for 2 days did not exhibit sustainability of cancer stem like cells features for tumor growth.	[119]
	MWCNTs	—	SiRNA and DNA	Internalization of MWCNTs by microglia: possible application in immunotherapy of brain tumors.	BV2 microglia and GL261 glioma cells	Uptake of MWCNTs by both BV2 and GL261 cells in vitro without any significant signs of cytotoxicity.	[120]

Blood cancer	SWCNTs	Sgc8c aptamer	Daunorubicin (Dau)	Reversible targeting and controlled release delivery of daunorubicin to cancer cells by aptamer-wrapped CNTs	Human T cell leukemia cell MOLT-4 and U266 myeloma cells	The tertiary complex Dau-aptamer-SWCNTs was internalized effectively to MOLT-4 cells but not to U266 cells and is less toxic in U266 as compared to Dau-aptamer-SWCNTs complex is able to selectively target MOLT-4 cells	[121]

Breast cancer	SWCNTs	Polyethylene glycol (PEG) and Poly (maleic anhydride-alt-1-octadecene) (PMHC_18_)	—	Optimization of surface chemistry on single walled CNTs for in vivo photothermal ablation of tumors	Balb/c mice bearing 4T1 tumors	PEG-PMHC_18_-SWCNTs showed ultralong blood circulation half-lives though showing high uptake in the tumor tend to accumulate in the skin dermis	[122]
	MWCNTs	Polyamidoamine dendrimer(d)	FITC-labelled antisense c-myc oligonucleotides (asODN)	Synthesis and characterization of polyamidoamine dendrimer coated MWCNTs and their application in gene delivery systems	MCF-7 and MDA-MB-435 human breast cancer cell line, liver cancer cell HepG2	Laser confocal microscopy confirmed the entry of asODN into the tumor cell, within 15 min of incubation. asODN-dMNTs composites inhibit the cell growth and downregulated the expression of the c-myc gene and C-Myc protein.	[123]
	SWCNTs	Insulin-like growth factor 1 receptor (IGF 1R-) specific and nonspecific monoclonal antibodies (mABs)	—	Bioconjugated CNTs for targeting cancer biomarkers	BT-474 and MCF-7 breast cancer cells	mAB-CNT as field effect transistors are very promising biosensor candidates to detect cancer cells.	[124]
	SWCNTs	Distearoylphosphatidy-lethanolamine (DSPE)-PEG-Amine, mouse double minute 2 homolog (MDM2)	SiRNA	Functionalized SWCNTs enables efficient intracellular delivery of SiRNA targeting MDM2 to inhibit breast cancer cells growth	B-Cap-37 breast carcinoma cells	f-SWCNTs showed significant efficiency in carrying SiRNA and SiRNA-MDM2 complexes in B-Cap-37 cells and caused inhibition of proliferating cells.	[125]
	SWCNTs	—	—	SWCNT nanobomb agents for killing breast cancer cells.	Human BT-474 breast cancer cells	SWCNT-based systems were capable of killing cancer cells without causing toxicity to the surrounding cells.	[126]
	SWCNTs	Polyethylene glycol	Paclitaxel (PTX)	Drug delivery with CNTs for in vivo cancer treatment	4T1 murine breast cancer cell line	SWCNT-PTX affords higher efficacy in suppressing tumor growth without causing obvious toxic effects to normal organs.	[110]
	SWCNTs	anti-HER2 chicken IgY antibody	—	Anti-HER2 IgY antibody-functionalized SWCNTs for detection and selective destruction of breast cancer cells	HER2-expressing SK-BR-3 cells and HER2-negative MCF-7 cells	Raman signal collected at single-cell level from the complex-treated SK-BR-3 cells was significantly greater than that from various control cells. HER2 IgY-SWCNT complex specifically targeted HER2-expressing SK-BR-3 cells but not receptor-negative MCF-7 cells.	[78]

Colon cancer	MWCNTs	—	—	Vertically aligned MWCNTs to preferentially entrap highly metastatic cancer cells	SW-48 cells and HT-29 human colon adenocarcinoma cell	Vertically aligned MWCNT entrap higher metastatic cancer in larger fraction than lower metastatic grades. Cell rigidity due to fixation decreases the entrapment efficiency	[127]

Colon cancer	SWCNTs	Bovine serum albumin-antibody, fluorescein	Doxorubicin	Triple functionalization of SWCNTs with doxorubicin, a monoclonal antibody and a fluorescent marker for targeted cancer therapy	WiDr Human colon adenocarcinoma cells	The triple fictionalized complex was efficiently taken by cancer cells with subsequent intracellular release of doxorubicin.	[128]

Liver cancer	MWCNTs	Polyamidoamine dendrimer(d)	FITC-labelled antisense c-myc oligonucleotides (asODN)	Synthesis and characterization of polyamidoamine dendrimer coated MWCNTs and their application in gene delivery systems	HEP-G2 human hepatoma cells	Laser confocal microscopy confirmed the entry of asODN into the tumor cell, within 15 min of incubation. asODN-dMNTs composites inhibit the cell growth and downregulated the expression of the c-myc gene and C-Myc protein.	[123]
	SWCNTs	Chitosan and folic acid	Doxorubicin	Targeted DDS based on chitosan and folic acid modified SWCNTs for controllable loading/release of anticancer agent doxorubicin.	Hepatocellular carcinoma SMMC-7721 cell lines	The chitosan-folic acid modified SWCNTs not only kill cancer cells efficiently, but also display much less in vivo toxicity than free doxorubicin.	[129]

Lymph node Metastatis	MWCNTs	Magnetic	Gemcitabine (GEM)	Magnetic functionalized CNTs as drug vehicles for cancer lymph node metastasis treatment	BxPC-3 pancreatic cancer cells	mMWCNTs-GEM had high antitumor activity resulting in successful regression and inhibition of lymph node metastasis under the magnetic field.	[130]

Kidney cancer	SWCNTs	—	—	Effect of SWCNTs on human HEK293 cells	Human kidney embryo cell HEK-293 cells	SWCNTs inhibit HEK293 cell proliferation and decrease cell adhesive ability in a dose and time dependent manner.	[131]
	SWCNTs	Phenosafranine (PS) and Nile blue (NB) dyes	—	SWCNTs modified with organic dyes: synthesis, characterization, and potential cytotoxic effects	Baby hamster kidney fibroblast cells BHK-21 cell line	Cytotoxicity of dye modified SWCNT displayed low toxicity in the dark while being higher in the dark and higher in the presence of light	[132]

Cervical cancer	SWCNTs	chitosan and folic acid, FITC	—	Functional SWCNTs chitosan conjugate for tumor cell targeting	Human cervical carcinoma HeLa cells	Conjugates provide new options for targeted drug delivery and tumor cell sensing because of the combined intrinsic properties of CNTs and the versatility of chitosan	[133]
	MWCNTs	Folate and iron	Doxorubicin	Folate and iron defunctionalized MWCNTs as dual targeted drug nanocarrier to cancer cells.	HeLa cells	This dual targeted nanocarrier has efficient biological and magnetical targeting capabilities towards HeLa cells and considered safe for biological applications.	[134]

Cervical cancer	SWCNT, MWCNT	Acid-treated SWCNTs, acid-treated MWCNTs, amylose wrapped SWCNTs.	—	The influence of CNT scaffolds on human cervical carcinoma HeLa cells viability and focal adhesion kinase expression.	HeLa cells	Cells cultured on SWCNTs and on acid-treated SWCNTs were found to undergo apoptosis. The cells cultured on MWCNTs, acid-treated MWCNTs, and amylose wrapped SWCNTs were found to be viable. This may be due to focal adhesion kinases (FAK) expression which is responsible for controlling the cell viability	[135]
	SWCNTs	Polysaccharides [sodium alginate (ALG) and chitosan (CH)]	Doxorubicin (DOX)	Targeted delivery and controlled release by doxorubicin to cancer cells using modified CNTs.	HeLa cells	The DOX released from the modified nanotubes has been shown to damage nuclear DNA and inhibit the cell proliferation.	[136]

Prostate cancer	SWCNTs	Polyethylenimine (PEI), Asn-Gly-Arg (NGR) peptide	SiRNA	Synergistic anticancer effect of RNAi and photothermal therapy mediated by functionalized SWCNTs.	Human prostate carcinoma (GIV) cell PC-3 cells	SWCNT-PEI-SiRNA-NGR induces severe apoptosis and suppresses the proliferating of PC-3 cells without any level of toxicity	[137]
